# Assessing the Nursing and Midwifery Students Competencies in Communication With Patients With Severe Communication Problems

**Published:** 2014-06-20

**Authors:** Mohsen Adib Hajbaghery, Zahra Rezaei Shahsavarloo

**Affiliations:** 1 Department of Medical-Surgical Nursing, Faculty of Nursing and Midwifery, Kashan University of Medical Sciences, Kashan, IR Iran

**Keywords:** Nursing, Midwifery, Students, Competency-Based Education, Patients, Hearing Loss

## Abstract

**Background:**

Clients with communication impairment are at risk for health disparity. Hence, health care workers should be knowledgeable and skillful in communication. However, no studies are available on Iranian nursing and midwifery students’ communication skills with patients with severe communication problems.

**Objectives:**

The present study was conducted to investigate Iranian nursing and midwifery students' competencies in communication with patients with severe communication problems.

**Materials and Methods:**

This study was performed on all senior nursing and midwifery students of Kashan University of Medical Sciences in spring 2013. Data were collected through a knowledge questionnaire and two checklists for evaluation of skills needed for communication with patients with severe communication problems. Data analysis was performed through independent samples t test, and Fisher’s exact test.

**Results:**

In total, 68.8% of the participants were female, 37.6% had a history of part-time job as a nurse or midwife. The mean score of knowledge were 4.41 ± 1.42 and 4.77 ± 1.77 for nursing and midwifery students, respectively and the difference was not significant (P = 0.312). In addition, the mean score of communication skills with deaf patients was 13.23 ± 4.68 and 11.86 ± 5.55 for nursing and midwifery students, respectively and the difference was not significant (P = 0.258). Also, the mean score of communication skills with stutter patients was 23.91 ± 4.17 and 21.25 ± 3.91 for nursing and midwifery students, respectively but the difference was not significant (P = 0.269).

**Conclusions:**

Nursing and midwifery students did not significantly differ in terms of communication with patients with severe communication problems. Most of the students had low or very low knowledge and skills in communication with patients with hearing impairment. However, they had better skills in communication with patient with speech problem. Special workshops or training programs are recommended to empower nursing and midwifery students in communication with patients with communication problems.

## 1. Background

Effective communication between health care providers and their clients is increasingly recognized as a crucial factor in patient-centered care (1, 2). Through communication, health care workers can identify clients’ needs and take appropriate actions to overcome their problems (3). However, it is difficult for nurses and other caregivers to work with clients with sever communication impairments such as hearing loss or stutter and other speech problems (4).

A communication disorder is defined as any disorder that affects somebody's ability to communicate. In such condition, the individual cannot meet its communication needs temporarily or permanently (5). Hearing and speech impairment are common communication disorders (6). In the United States (U.S), 13% of people have hearing difficulties (7). In addition, it is reported that at least one-third of the elderly experience some degree of hearing impairment (8). The magnitude of speech impairment in Iran is not known. However 1% of the general population and about 9% of young children in the U.S suffer speech impairments (9, 10). Clients with communication impairment are usually dependent on their close relatives to communicate with health care providers; however, relatives may sometimes interfere with the treatment process (11, 12). These clients are at risk for health disparity and usually report lower health status than the general population (13). They also feel great anxiety in medical visits and therefore might feel that they do not receive appropriate care (7, 14, 15).

A significant number of clients who need nursing care are unable to speak because of acquired or developmental disabilities. Moreover, some clients are temporarily unable to speak when they are in hospital because of intubation or some neuromuscular disorders such as Guillian-Barre Syndrome (4).

Health care organizations should have staffs capable of communicating with patients having communication impairment (16). Park and Song studied the communication barriers perceived by the elderly clients and nurses. They reported that clients’ hearing problems were the main communication barriers between nurses and the elderly clients (17). Ralston et al. also reported that many physicians and health care workers misjudged the intelligence of people with communication impairment. Such attitude then might affect their conduct with such people (18). Many health care workers also think that people with hearing impairment are able to lip-read while most people with hearing difficulties have not acquired lip-reading skills. Then the lack of staff skilled in communication with the patients with hearing impairment, exaggerates the problems in communication between health care providers and patients with hearing problems (12).

Nursing and midwifery students should have high levels of communication skills (19). Studies have shown that nursing and midwifery schools do not effectively teach communication skills. Hence, nursing and midwifery students have difficulties in communicating with patients with normal hearing and speech (20, 21). In addition, Taghizadeh et al. investigated communication skills of midwives and reported that more than 50% of midwives were deficient in their verbal and nonverbal communication skills (2).

A number of investigators have studied the medical and nursing staff in communicating with patients with hearing and visual disabilities (7, 12). Some studies have also studied nursing students’ communication skills (20) however, they did not focus on communication with clients with hearing and speech impairment.

## 2. Objectives

The present study was conducted to investigate the nursing and midwifery students' competencies in communication with patients with hearing and speech impairment.

## 3. Materials and Methods

A cross-sectional study was performed on all nursing and midwifery students before graduation from Kashan Nursing School in spring 2013. A census sampling method was applied and the inclusion criteria were being a last year student and willingness to participate in the study. Data collection instrument was designed through literature review (7, 12, 14, 22). A five part instrument was used in this study. The first part of the instrument contained questions regarding age, gender, semester, history of part-time job as a nurse or midwife, previous training on communication with people with deafness or speech disorders.

The second part was a knowledge questionnaire and consisted of 10 multiple-choice questions on communication with patients with severe hearing impairment (seven items) and stutter (three items). Every question had a correct answer that scored as “one”. Therefore, the score of knowledge was between zero to 10. Obtaining a score between nine to ten was considered as high level knowledge, and then scores eight to seven, six to five and four or lower were considered as average, low, and very low knowledge, respectively.

The third part of the instrument was a checklist for assessment of skills in communication with a patient with stutter. This checklist consisted of 13 items. The first item was in Yes = 2 or No = 0 format and assessed the students skill in starting communication. The next 11 items were on the subjects' skill during communication. Two out of the 11 items were in Yes = 2 or No = 0 format and the other 9 items were in four-choice Likert scale from “yes, throughout the session = 3” to “never was done = 0”. The last item assessed the subjects' skill on receiving feedback from the simulated patient and scored as the aforementioned 11 items. The total score in this checklist ranged from 37 to zero. Obtaining scores were categorized as high (37-33), average (32-26), low (25-18) and very low (17-0).

The fourth part of the instrument was a checklist for assessment of skills in communication with a patient with hearing impairment and consisted of 15 items. This checklist also evaluated the student’s behavior in three domains of “beginning of communication”, “during communication”, and “receptive communication”. The first section had four dichotomous items (Yes = 2 or No = 0) with a total score from zero to eight. The second section had nine items scored in a four-choice Likert scale from “yes, throughout the session = 3” to “never was done = 0”. The total score in this section ranged from zero to 27. The third section that was about receptive communication evaluated the subjects' skills on receiving feedback from the simulated patient. This part had two items whose scoring was similar to the second part and its total score ranged from zero to six. The overall score of this checklist ranged from zero to 41. The total scores were categorized as high (41-35), average (34-29), low (28-21) and very low (20-0). A choice of “I do not understand” was included in all items of the two checklists. If the observer was uncertain about the occurrence of a behavior, this option was checked; then the film of session was reviewed and the appropriate option was marked.

Content validity of the knowledge questionnaire was confirmed by seven nurse instructors, an audiologist and a speech therapist. Reliability of the knowledge questionnaire was confirmed through split-half method after administering on 20 nursing students. The Pearson correlation coefficient of the test halves was calculated (r = 0.76). Content validity of the skill assessment checklists was confirmed by seven nurse instructors, a speech therapist and an audiologist. The reliability of skill assessment checklists was confirmed through inter-raters' reliability. Therefore, the second researcher and a clinical instructor concurrently observed 20 nursing students in clinical setting and completed the checklist. Then the Kappa agreement coefficient was calculated and ranged from 0.6 to one for different items. In addition, Cronbach's alpha coefficient was calculated to assess the internal consistency of the two checklists and were 0.70 and 0.75 for the speech and hearing checklists, respectively. The fifth part of the instrument was a questionnaire about the students' attitude toward the methods used in this study. This part of the instrument was adopted from the questionnaire previously developed by Muldoon et al. (23). Content validity of this part was confirmed by the seven nursing instructors. The reliability of this part was previously assessed by Muldoon et al. using test re-test method and the Spearman's correlation co-efficient was 0.871 (23).

### 3.1. Observation Setting

The students were evaluated in three stations. At the first one, students performed a short interview with a simulated patient who portrayed a patient with deafness and elementary education who experienced pain in his chest. At the second one, students performed a short interview with a female simulated patient who portrayed a patient with stutter and diploma education who experienced erythema and pruritus in her hands from a day before. Finally the students entered the last station in which they completed the knowledge, demographic and attitude questionnaires. The second author was seated in the corner of the first station and assessed the students’ skills. A co-researcher that was previously trained and tested performed the skill assessment in the second station.

All students were initially quarantined and briefed on what they were anticipated to do. The direction for each station was posted on the station door and informed the students about the station structure, its content, and the expected tasks. The stations were close to the quarantine and the time of each station was ten minutes. At the first station, the simulated patient was on his bed, back to the door. In the speech station the simulated patient entered a few second after the student entered the station. The simulated patient and the evaluator in each station were identical for all students. In addition, a hidden camera recorded all the sessions.

### 3.2. Ethical Considerations

The University Review Board and Research Ethics Committee at Kashan University of Medical Sciences approved this study. The objectives of the study and existence of a hidden camera were explained to all participants. They were all assured of the privacy of their personal information and signed an informed consent form before participating in this study.

### 3.3. Data Analysis

Data analysis was done by SPSS version 11.5 (SPSS Inc. Chicago, Illinois). The level of statistical significance was selected to be lower than 0.05. Descriptive statistics were used. Moreover, independent samples T test was used to examine the differences in knowledge and skills scores in the two groups. In addition, Fisher’s exact test was used to compare the levels of knowledge and skills in nursing and midwifery students.

## 4. Results

Out of 95 students, two students were excluded due to the lack of consent and finally 93 students were enrolled. In total, 71 (76.3%) and 22 (23.7%) participants were nursing and midwifery students, respectively. Mean age of the students was 22.95 ± 8.32 years. In total, 68.8% of the participants were female, 37.6% had a history of part-time job as a nurse or midwife. Only 2.2% of students had been trained on communication with people with deafness and 3.2% had been trained on communication with people with speech disorders (out of the university).

None of the subjects had a high level knowledge and skills in communication with patients with deafness while more than one third and about half of the students in both groups showed high level knowledge and skills in communication with patients having speech disorder, respectively. Moreover, more than 90% showed a low or very low skill in communication with patients with deafness (Table 1).

**Table 1.** The Levels of Knowledge and Skills Among Nursing and Midwifery Students^a,b,c^

Variables	High	Average	Low	Very low	P value
**Knowledge (Total score)**					0.795
Nursing	0	4 (5.7)	31 (44.3)	35 (50.0)	
Midwifery	0	2 (9.1)	10 (45.5)	10 (45.5)	
**Knowledge of communication with deaf patient**					0.403
Nursing	0	1 (1.4)	26 (37.1)	43 (51.4)	
Midwifery	0	0	12 (54.5)	10 (45.5)	
**Knowledge of communication with stutter patient**					0.533
Nursing	28 (39.4)	28 (39.4)	13 (18.3)	2 (2.8)	
Midwifery	8 (36.4)	12 (54.5)	2 (9.1)	0	
**Communication skills with deaf patients (Total score)**					0.622
Nursing	0	0	4 (5.6)	67 (94.4)	
Midwifery	0	0	1 (4.5)	21 (95.5)	
**Communication skills with Stutter patients (Total score)**					0.464
Nursing	34 (47.9)	24 (33.8)	12 (16.9)	1 (1.4)	
Midwifery	11 (50.0)	10 (45.5)	1 (4.5)	0	

^a^ Data are presented as No. (%).

^b^ Fishers’ exact test was performed.

^c^ One student in each group was excluded due to incomplete responses to the knowledge questionnaire

The total mean score of knowledge were 4.41 ± 1.42 and 4.77 ± 1.77 for nursing and midwifery students, respectively and the difference was not significant (P = 0.312). The total mean score of communication skills with deaf patients was 13.23 ± 4.68 and 11.86 ± 5.55 for nursing and midwifery students, respectively but the difference was not significant (P = 0.258). In addition, no significant difference was found between the two groups in the mean scores in skills subscales (Table 2).

Moreover, the total mean score of communication skills with stutter patients was 23.91± 4.17 and 21.25 ± 3.91 for nursing and midwifery students, respectively but the difference was not significant (P = 0.269). Also, a significant difference was found between nursing and midwifery students in the subscale of "start of communication" so that the mean score was higher in nursing students (Table 2).

**Table 2.** Mean and Standard Deviation of Knowledge and Skills of Nursing and Midwifery Students^a^

Items	Nursing	Midwifery	P Value
**Knowledge and skills in communication with deaf patients**			
**Knowledge**	2.24 ± 1.17	2.43 ± 1.43	0.548
**Skills in start of communication**	2.17 ± 1.25	1.82 ± 0.85	0.622
**Skills in during communication**	12.65 ± 3.81	11.59 ± 4.83	0.331
**Skills in receptive communication**	1.04 ± 1.08	0.95 ± 1.04	0.740
**Skills (Total score)**	13.23 ± 4.68	11.86 ± 5.55	0.258
**Knowledge and skills in communication with Stutter patients**			
**Knowledge**	2.16 ± 0.81	2.22 ± 0.68	0.761
**Skills in start of communication**	0.33 ± 0.75	0	0.001
**Skills in during communication**	21.29 ± 2.74	20.45 ± 2.10	0.164
**Skills in receptive communication**	2.32 ± 0.67	2.45 ± 0.73	0.190
**Skills (Total score)**	23.91 ± 4.17	21.25 ± 3.91	0.269

^a^ Data are presented as Mean ± SD

The students’ attitude toward the testes and stations used in the current study are presented in Table 3. The majority (58.06%) of the students reported that the manner used in this study was a meaningful way for assessing their clinical skills. Also the majority (50.54%) of them reported that the stations could reflect real-life clinical conditions. Only 20.43% of the students believed that the stations were stressful while more than 50% reported that the method used could be helpful in increasing their confidence in clinical practice.

**Table 3.** The Students Attitude Toward the Methods Used in the Current Study

The Methods Used in This Study	Agree	Neutral	Disagree	Not Responded
**Was a meaningful way for assessing my clinical skills**	54 (58.06)	26 (27.96)	12 (12.90)	1 (1.08)
**Reflected real-life clinical conditions**	47 (50.54)	32 (34.41)	14 (15.05)	0
**Was a fair method of assessment**	9 (9.68)	30 (32.26)	54 (58.06)	0
**Provided me with an opportunity to show my clinical knowledge**	36 (38.71)	41 (44.09)	16 (17.20)	0
**Provided me with an opportunity to show my practical skills**	35 (37.63)	34 (36.56)	23 (24.73)	1 (1.08)
**Took placed in a suitable environment**	54 (58.06)	28 (30.11)	11 (11.83)	0
**Is helpful in increasing the students' confidence in**	47 (50.54)	28 (30.11)	18 (19.35)	0
**The examiner made me feel at ease**	30 (32.26)	33 (35.48)	26 (27.96)	4 (4.30)
**Guidelines I got were helpful for preparation**	43 (46.24)	32 (34.41)	17 (18.28)	1 (1.08)
**The length of each station (i.e. 10 min) was sufficient**	36 (38.71)	28 (30.11)	28 (30.11)	1 (1.08)
**I did not understand the purpose of the stations**	11 (11.83)	18 (19.35)	63 (67.74)	1 (1.08)
**The skills being evaluated in the stations were not reflective of those required in clinical practice**	18 (19.35)	36 (38.71)	38 (40.86)	1 (1.08)
**I did not feel prepared for the stations**	32 (34.41)	34 (36.56)	27 (29.03)	0
**I was not very nervous during the stations**	45 (48.39)	17 (18.28)	30 (32.26)	1 (1.08)
**I found the stations very stressful**	19 (20.43)	22 (23.66)	52 (55.91)	0
**The stations has benefited me in developing my knowledge and skills**	45 (48.39)	36 (38.71)	12 (12.90)	0
**I was confident during the stations**	42 (45.16)	34 (36.56)	17 (18.28)	0

## 5. Discussion

The present study showed that most nursing and midwifery students had a low to very low knowledge and skills in communication with patients having severe communication problems. These findings were consistent with previous studies. In a study of the relationship between patients with deafness and the doctors, it has been reported that health care team was not ready to care for patients with deafness and it would put patients at risk for misdiagnosis and inappropriate treatment (24). Another study reported that women with deafness had lower levels of health information and had used less preventive health care such as pap smear and mammography than other people (13). Although no studies are available about problems of people with speech disorders in the health care system, Snow and Powell has studied the importance of oral language competence and reported that people with severe speech difficulties have encounter many problems in police interviews and court processes (25). Castles also investigated the problems in communication between nurses and people with hearing impairment and reported that nurses had inadequate skills in communication with such clients. Based on the Castles’ report, the nurses’ inability to evaluate the effects of medications, difficulties in patient training, and taking the patients consent for therapeutic interventions were among the most prevalent problems in nurses communication with these patients (26).

The present study showed that none of the nursing and midwifery students had high level knowledge on communication with people with deafness. In addition, the both groups did not significantly differ in this regard. In a study only 10% of nurses expressed that they had received sufficient training on developmental disabilities (27). In a study on communication between nurses and patients with severe communication impairment, only one nurse reported that has got some information on communication with patients having speech disorders from a speech pathologist. According to Hemsley et al. most nurses usually overcome their communication problems with helps from a speech pathologist, family members or other nurses (4).

While no one in the present study had good knowledge and skills in communication with patients having hearing loss, more than one third of students showed a high level knowledge and about half of them had a high level skills in communication with the simulated patients with stutter. Although no studies are available on nursing or midwifery students' problems in communication with patients having speech problems, nurses in a previous study reported that they need increased support to facilitate an effective communication with these patients (4). The better communication of the students with patients having speech disorders can be attributed to the fact that these patients could receive the students’ messages through hearing and only had difficulty in feedback while the deaf or hearing impaired patient have difficulties both in receiving the messages and in returning back their feedback. Then, nurse-patient communication is harder both for nurses and patients with severe hearing impairment.

In the present study most of the students expressed positive attitude toward the evaluation method used in the study. They reported that this method was an appropriate way for assessing the students' clinical skills. It seems that using simulated patients is an applicable, satisfactory and reliable method for both the students and their instructors. Then using the same method in evaluating the nursing and midwifery students is suggested.

The current study revealed that nursing and midwifery students were lacking in knowledge and skills required for effective communication with patients who were deaf or had speech impairment. This problem may be related to the content of nursing and midwifery curricula. Then nursing and midwifery education system should pay more attention to this issue as it is responsible to prepare competent nurses for the common issues they would face in practice.

Some limitations may limit the generalizability of results. The number of the subjects in this study was limited as the study conducted in a nursing and midwifery school. Therefore, a study with larger samples from different schools is recommended. Although we used a simulated patient, using real patients or observing the students' conduct in a real clinical setting is suggested. In addition, we tried to prevent any bias from the side of the research team; however, the possibility of evaluator bias may be considered as a limitation of the study. It is also suggested that the attitude of nursing and midwifery students toward patients having complex communication needs be assessed.
